# Characterization of Homicides in Mexico: Analysis of 2015–2022

**DOI:** 10.3390/ijerph21050617

**Published:** 2024-05-13

**Authors:** Eduardo López-Ortiz, Juan Manuel Altamirano, Luisa Fernanda Romero-Henríquez, Geovani López-Ortiz

**Affiliations:** 1 Subdivisión de Medicina Familiar, Facultad de Medicina, Universidad Nacional Autónoma de México, Mexico City 04510, Mexico; eduardolptz@gmail.com; 2 Departamento de Neurocirugía, Hospital Ángeles Clínica Londres, Mexico City 06700, Mexico; jmaltamirano83@gmail.com; 3 Posgrado en Pedagogía, Facultad de Filosofía y Letras, Universidad Nacional Autónoma de México, Mexico City 04510, Mexico; fernandaromero55@yahoo.com.mx; 4 Cuerpo Académico CAMCM-CA-2321, Centro de Actualización del Magisterio en la Ciudad de México, Autoridad Educativa Federal, Secretaría de Educación Pública, Mexico City 06400, Mexico

**Keywords:** violence, homicides, deaths, Mexico

## Abstract

Background: In Mexico, homicides are the leading cause of death among men aged 15 to 44 years; however, despite their increase in recent decades, the study of this issue is insufficient, given its magnitude and impact. Therefore, this study aimed to characterize the spatial and temporal patterns and associated factors of homicides in Mexico from 2015 to 2022. Methods: An analytical cross-sectional study was conducted, analyzing death records from the National Institute of Statistics and Geography’s general mortality databases. Simple frequencies and incidence rates per 100,000 inhabitants by sex, year, and state of the Mexican Republic were calculated. Mortality was evaluated by age groups and geographic areas, and bivariate logistic regression models with sociodemographic variables were performed. Results: Records of 229,182 homicides in Mexico were analyzed, with a median age of 33 years, interquartile range 18. A total of 203,898 (88.96%) were men and 25,284 (11.04%) were women. The majority of deaths occurred in public places and were caused by firearms; women had a higher percentage of homicides at home. States with high incidence rates for both sexes were Chihuahua, Zacatecas, Michoacán, Colima, and Estado de México. The total years of life lost were 9.19 million years. The national incidence of homicides in men showed an upward trend from 2015 to 2019; however, in the case of women, this incidence increased in various age groups during the study period. Occupation, education, marital status, and place of occurrence had significant associations in the logistic regression models. Conclusions: This study provides a spatial-temporal characterization of homicides in Mexico between 2015 and 2022, highlighting the high incidence in men and the upward trend in certain age groups among women. These findings underscore the need for preventive measures and public policies to address this issue in a multisectoral manner.

## 1. Introduction

Violence, particularly homicides, is often assumed to be a judicial problem. Its repercussions have led it to be considered by the World Health Organization (WHO) as a critical public health issue that extends beyond the mere act of aggression, affecting communities, societies, and nations at large. Due to its magnitude, its reduction has been proposed as part of the agenda towards 2030 for sustainable development [1,2,3,4].

The recognition of violence as a public health concern is not novel; however, its persistent prevalence, particularly in Mexico, necessitates a renewed focus on its etiology, consequences, and mitigation strategies [5,6]. International organizations, including the United Nations (UN) and the WHO, have long advocated for an intersectoral approach to address violence [4,7].

Such strategies emphasize the importance of collaborative efforts that span governmental, non-governmental, and civil society sectors, aiming to implement comprehensive and sustainable interventions that address the root causes of violence, including socio-economic inequalities, lack of educational and employment opportunities, and systemic injustices [8,9].

The burden of homicides and violence disproportionately affects the most vulnerable populations within societies in a scenario in which the region of the Americas represents 33% of homicides worldwide, which makes it one of the regions with the highest rates of homicides [10,11]. Studies have consistently shown that individuals and communities at the socio-economic margins are at a heightened risk of experiencing violence [12,13,14].

In Mexico, the incidence of homicides has alarmingly increased over the last two decades, becoming one of the leading causes of death among men. Concurrently, the number of female homicide victims has risen by approximately 50% during the period from 2013 to 2022 [15]. These deaths directly impact life expectancy at birth for both genders and affect the country’s demographic dynamics [11,16].

While various approaches have been taken to explain the causes of homicides in the country, there is a lag in understanding the factors that contribute to their occurrence, such as structural and sociodemographic causes, including poverty, lack of education and marginalization, that increase the vulnerability of certain groups to becoming victims of homicide. This context highlights how violence, far beyond being a security issue, represents a serious public health problem closely linked to broader social inequalities affecting Mexico [15,17,18,19].

Understanding the epidemiological patterns of homicides within this context requires a nuanced analysis of the interplay between individual, community, and societal factors. Moreover, it calls for an examination of the effectiveness of existing interventions and the development of innovative strategies to combat this public health menace [20,21,22,23].

In investigating mortality by homicides in Mexico from 2015 to 2022, this article aims to contribute to the burgeoning discourse on violence as a public health issue by examining the spatiotemporal trends within Mexico to identify key characteristics of the profile of the most vulnerable groups.

## 2. Materials and Methods

### 2.1. Study Description

An analytical cross-sectional study was conducted. Death records classified and reviewed as homicides (excluding accidental deaths and suicides) according to the procedures established by regulations in Mexico from the general mortality databases of the National Institute of Statistics and Geography (INEGI) from 2015 to 2022 were examined [24]. Records of homicides caused by gunshot wounds, injuries from sharp objects, strangulation, drowning, and physical or chemical means, among others, were analyzed. Inclusion criteria were confirmation of sex, state of occurrence and age. Sociodemographic information about marital status, education level, occupation, place of death, life expectancy, state poverty percentage, and municipal marginalization degree was included for analysis. Records incomplete or lacking information regarding the federal entity of occurrence and sex were excluded.

Different causes of homicides were classified according to the mechanism and place of occurrence for both sexes, and the different federal entities where the deaths were registered were identified. Likewise, simple frequencies and incidence rates per one hundred thousand inhabitants were calculated for each sex using the estimated projected population by the National Population Council of Mexico (CONAPO) [25]. Years of life lost were calculated using life expectancy by state calculated by INEGI [26].

Mortality by age groups and zones was evaluated by classifying the federal entities into five regions according to social mobility characteristics, including access to health care, education, and the labor market. These regions were: (1) North: Baja California Norte, Coahuila de Zaragoza, Chihuahua, Nuevo León, Sonora, Tamaulipas. (2) Northwest: Baja California Sur, Durango, Nayarit, Sinaloa, Zacatecas. (3) Central: Mexico City, Guanajuato, Hidalgo, State of Mexico, Morelos, Puebla, Querétaro, Tlaxcala. (4) North Central: Aguascalientes, Colima, Jalisco, Michoacán de Ocampo, San Luis Potosí. (5) South: Campeche, Chiapas, Guerrero, Oaxaca, Quintana Roo, Tabasco, Veracruz de Ignacio de la Llave, Yucatán [27].

Multidimensional analysis of poverty and CONAPO marginalization indices were used to describe social vulnerability in states with the highest homicides and highest incidence rates [28].

Prevalences by federal entity were calculated for the following sociodemographic factors: occupation, education level, marital status, and place of occurrence.

### 2.2. Statistical Analysis

For the calculation of incidence rates and their confidence intervals by age group, Bayesian inference implemented in the Stan programming language included in the Surveil library of the R statistical environment using the R Studio interface version 4.4.2 was used [29].

We conducted a bivariate logistic regression analysis using a dichotomous dependent variable that included all causes of homicide in contrast to all other causes of death, including those that were not homicide, like accidental deaths or suicides, to investigate the impact of sociodemographic and structural factors; the models were run by sex. The model specifically aimed to assess how these characteristics and the sex of the individual influenced the likelihood of dying by homicide. Reference categories were selected according to the least vulnerable as reported in the literature, or the least prevalent in the descriptive analysis. This approach allows us to understand not just the effect of each variable on its own but also how the impact varies by sex. Following the model fitting, odds ratios with 95% confidence intervals were computed for each predictor to facilitate interpretation of the effects in terms of the likelihood of the outcome occurring. To calculate years of life lost, the age at death was subtracted from the life expectancy by state [30].

### 2.3. Ethical Considerations

Since patients or medical records were not involved, and information available on websites was used, approval from an ethics committee for this research was not required.

## 3. Results

The records of 229,182 homicides in Mexico were analyzed, with a median age of 33 years with an interquartile range (IQR) of 18 years. A total of 203,898 (88.96%) men and 25,284 (11.04%) women lost their lives, with the majority of deaths occurring in public places, accounting for 49.65% and 37.47% for men and women, respectively. Table 1 summarizes the sociodemographic characteristics at the national level.

**Figure 1:**
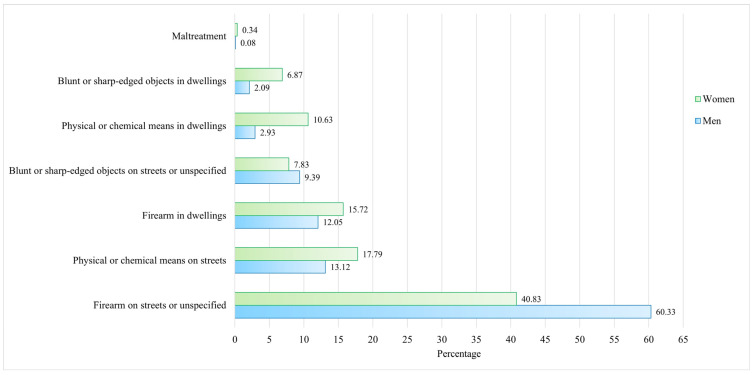
Causes of homicides in Mexico by sex according to the mechanism and place of occurrence (2015–2022).

When analyzing homicides according to the mechanism and place of occurrence by sex, it was identified that the majority of deaths occurred due to firearm use in streets or unspecified locations, accounting for 68.86% and 50.56% for men and women, respectively. Regarding homicides that occurred at home, women showed higher percentages, regardless of the mechanism through which these deaths were perpetrated (Figure 1).

Homicides in men represented 6.17% of the total deaths for this gender in the country. The states with the highest number of deaths were Baja California Norte, Chihuahua, Guanajuato, Estado de México, and Michoacán, accounting for 40.23% of all homicides recorded during the study period. On the other hand, Baja California, Colima, Chihuahua, Guerrero, and Zacatecas had a higher incidence rate per 100,000 men (Supplementary Materials, Table S1).

In the case of women, homicides accounted for 0.95% of the total deaths during the study period for this gender. Baja California Norte, Chihuahua, Guanajuato, Jalisco, and Estado de México were the states with the highest number of homicides, comprising 42.78% of the total. Baja California Norte, Colima, Chihuahua, Guanajuato, and Zacatecas had the highest incidence rates per 100,000 women (Supplementary Materials, Table S2).

**Figure 2:**
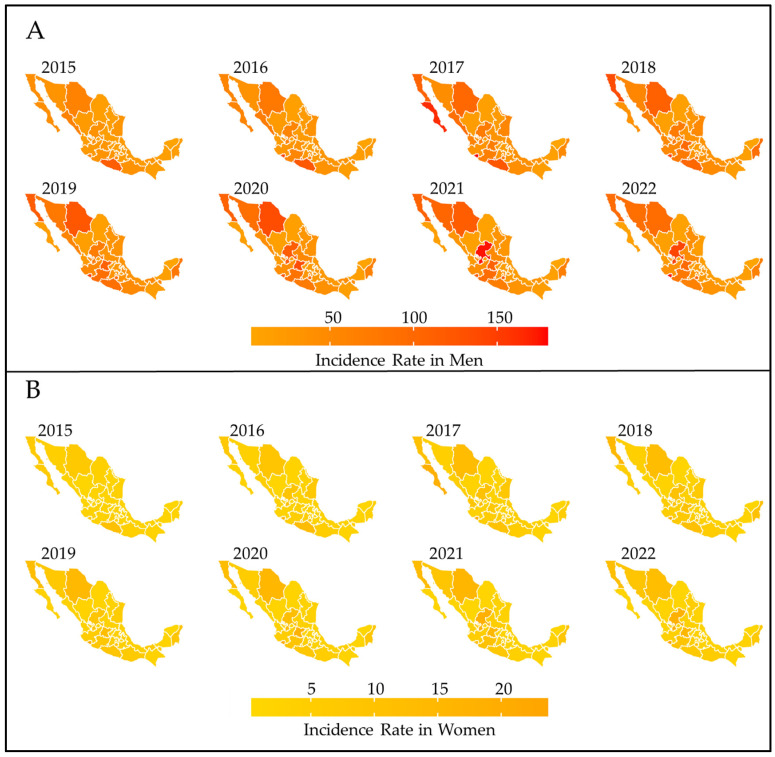
Choropleth map of the incidence of homicides by sex, by state from 2015–2022. (**A**) men, (**B**) women.

Regarding the spatiotemporal analysis of homicides distributed by sex, it was identified that starting from the year 2017, the northern region of the country experienced an increase in the incidence of homicides in men, as well as the states of Zacatecas, Guanajuato, and Morelos. In the case of women, the states of Baja California Norte, Sonora, Chihuahua, Zacatecas, Guanajuato, and Morelos maintained the highest incidence rates. Additionally, regions with high incidence rates for both sexes were Chihuahua, Zacatecas, Michoacán, Colima, and Estado de México (Figure 2).

During the study period, a total of 8,165,134.92 years of life were lost due to homicides in men, representing 18.72% of the total years of life lost for this gender. The five states with the highest number of homicides in men accounted for 3,340,445.22 years of life lost. In women, the total years lost due to homicides was 1,028,494.18, representing 6.91% of the total years of life lost for this gender throughout the period.

The average age of death due to violent causes was lower in the states of Guanajuato, Quintana Roo, San Luis Potosí, and Zacatecas in men, while in the case of women, it was lower in Colima, Chihuahua, and Zacatecas. An increase in age was associated with a lower probability of death by homicide in both sexes.

### 3.1. Mortality by Sex According to Age Groups

The three age groups that, on average, had the highest incidence per 100,000 men were 25 to 29 years, with 80.50 cases; 30 to 34 years, with 81.43; and 35 to 39 years, with 79.64. In the case of women, the three groups that, on average, had the highest incidence were 20 to 24 years, with 8.78 cases; 25 to 29, with 9.05; and 30 to 34, with 8.28. For both sexes, the regions with the highest number of deaths were North Central, North, and Northwest (Table 2 and Table S3).

**Figure 3:**
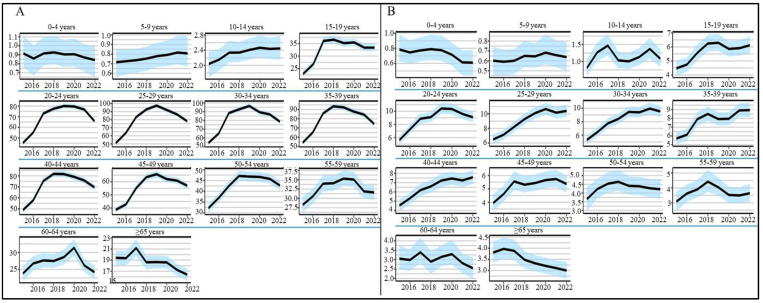
National incidence rate per 100,000 inhabitants of homicides in Mexico by age group and sex during the study period (2015–2022). (**A**) Men, (**B**) Women (*y*-axis: incidence; blue color: confidence level).

When analyzing the temporal trends in the national incidence of homicides per 100,000 men, there was an upward trend from 2015 to 2019 in most age groups. However, starting in 2020, decreases in incidence were recorded in all age groups except for children aged 0 to 14 years. On the other hand, for women, there was an increase in incidence from 2015 to 2022 in the age groups of 15 to 19 years and 25 to 44 years, with the ages of 15 to 19, 25 to 29, and 40 to 44 years being the most affected (Figure 3).

### 3.2. Sociodemographic Factors Associated with Homicides

Within states with higher violence indices, a heterogeneous profile of marginalization and poverty was identified. In four of the states with the highest number of homicides and in three with the highest incidence, the average municipal marginalization was lower than the national average. In contrast, the percentage of the population living in poverty was higher in states with a higher incidence than the national average. Furthermore, the percentage of the state population with educational lag in three of the five states with the highest number of deaths and incidence rates was higher than the national average. These structural factors, such as the higher percentage of marginalization, the percentage of poverty, and the percentage of state population with educational lag, had a higher probability of death by homicide, with differences among regions, especially in the south. The north was the most vulnerable due to the mentioned structural factors (Supplementary Materials, Table S4).

#### 3.2.1. Age

Considering the difference in frequency and incidence rate per 100,000 inhabitants of homicides between men and women (Table 2 and Table S3), we observe that the most vulnerable groups are structurally concentrated according to the percentages around the age groups from 15–19 years to 40–44 years. The probabilities of death by homicide were higher in the 20–25 years age group (OR 23.05, CI 20.89–25.51) for men and 20–24 in women (OR 8.78, CI 7.83–9.88) compared to those aged 0–4 years. The odds of homicide continue to increase in men up to the 55–59 years age group, whereas for women, the risk decreases from the age of 45 onwards.

#### 3.2.2. Occupation

In 26 states of the country, the highest percentage of men in administrative-related occupations had higher probabilities of death by homicide compared to people in agricultural-related occupations (OR 1.68, CI 1.64–1.71). Coahuila had the highest prevalence at 40%. In two states, agricultural-related labor was more prevalent, Oaxaca had the highest percentage at 40%, while in four states, the category with the highest prevalence was unspecified labor activity (OR 3.48, CI 3.43–3.54), with Baja California Norte accumulating 39%. Unemployment was the most prevalent category among women (40%) in all states of Mexico with lower odds of death by homicide (OR 0.13, CI 0.12–0.15) compared with those women involved in agriculture occupations. Of note, security-associated jobs were the only category with higher odds in women, while in men all categories except unemployed had higher probabilities of death by homicide.

#### 3.2.3. Education

In 28 out of 32 states, the most prevalent education level for homicides in men was secondary or high school (OR 2.70, CI 2.66–2.75) compared with men with a bachelor’s degree. Sonora had the highest percentage at 62%. In the remaining four states, the most prevalent education level was elementary or none with lower probabilities of death by homicide (OR 0.77, CI 0.76–0.79), with Michoacán having the highest prevalence at 48.17%.

For women, the most prevalent education level among homicide victims was secondary or high school in 29 out of 32 states. This group had higher probabilities of death by homicide (OR 1.74, CI 1.67–1.81) compared with women with a bachelor’s degree. In the remaining three states, the most frequent education level was elementary or none, which as seen in men, had the lowest odds of death by homicide (OR 0.29, CI 0.28–0.31). Oaxaca had the highest percentage, 47.84%.

#### 3.2.4. Marital Status

Marital status among male homicide victims was heterogeneous. In 20 states, the most prevalent status was married, with Oaxaca having the highest prevalence (63.98%). In 10 states, single individuals had a higher prevalence than married ones, with higher odds of death by homicide compared with married men (OR 2.7, CI 2.6–2.7), with Baja California having 66.55%. In the remaining two states, having no information on marital status was the most prevalent; this category also had higher odds of death by homicide compared to married men (OR 1.69, CI 1.67–1.73).

For women, marital status also had a heterogeneous distribution. In thirteen states, being single was the most prevalent status and the category with highest odds of death by homicide (OR 2.27, CI 2.17–2.34). Campeche had the highest prevalence (64.93%). On the other hand, being married was the most prevalent status in 17 states, with Baja California Norte presenting the highest prevalence (68.20%).

#### 3.2.5. Place of Occurrence

The most prevalent place of occurrence of homicides among men was streets and highways compared with private hospitals in 30 states (OR 41.78, CI 95% 40.25–43.39). Michoacán had the highest percentage at 59.67%. Yucatan and Baja California Norte were the remaining two states where the place of death occurrence was unknown (OR 12.75, CI 95% 12.28–13.25).

For women, in 19 states, streets and highways were the place with the highest odds of death by homicide (OR 113.29, CI 95% 121.89–150.67). Guanajuato had the highest prevalence (50.80%). In seven states, home was associated with higher odds of death by homicide (OR 1.5, CI 95% 1.35–1.68); Campeche had the highest prevalence (49.35%). This information was unknown in six states, with Baja California Norte having the highest prevalence at 48% with higher odds of death by homicide (OR 20.12, CI 18.13–22.41). Table 3 summarizes the aforementioned sociodemographic characteristics and their relationship with homicides.

## 4. Discussion

Violence is a public health issue that exhibits similarities to infectious epidemics, sharing risk factors related to social determinants, seasonality, clustering, and age groups with more significant vulnerabilities [31]. These characteristics position it as a complex challenge that requires a response integrating various sectors of society, multidisciplinary participation, and transgenerational commitment [32].

Violence disproportionately affects men more than women worldwide, with this gender difference being nearly double in Asia and nine times higher in the Americas region [10,33,34]. In our analysis, we found an average of 44 deaths per hundred thousand inhabitants among men, a figure higher than the estimated 15 deaths per hundred thousand inhabitants for the Americas region, and five homicides per hundred thousand inhabitants in women at the national level [10].

This asymmetry extends when we examine the eight-to-one ratio in estimated years lost and prevalence of homicides by sex, 6% in men and 0.9% in women, contrasted with the 2.27% reported by the WHO for the global population [35].

Moreover, we identified that the quantity of homicides is not directly related to the population size in a given area [36]. This suggests that the clustering profile of this phenomenon depends not only on population size but also on other structural spatiotemporal factors, such as poverty [37], degree of marginalization, and educational lag, and access to effective hospital healthcare (given a nearly 70% coverage, but increased risk of death in these network hospitals), all of which also showed significant associations in our analysis.

Considering the difference in the magnitude of homicides between men, women face unfavorable conditions reflecting social vulnerabilities characteristic of their sex [10]. For instance, in the analysis by age groups across all regions of the country, women die at a younger age compared to men, with their place of death often being unknown, and they exhibit the highest unemployment rates.

The conditions of unemployment in women and the higher proportion of states where their deaths occur among individuals with no formal education or those with primary education make it difficult for them to access job opportunities that reduce their vulnerability, leaving them exposed to environments of workplace and domestic violence [38,39].

On the other hand, individuals with secondary education face a higher likelihood of becoming victims of homicide compared to those with higher levels of education. Conversely, those with only primary education are less likely to experience violent deaths. This U-shaped correlation between educational level and the probability of death by homicide could be related to socio-economic factors and their association with exposure to violence. Individuals with secondary education might experience different socio-economic stresses or live in environments with higher crime rates compared to those with primary or higher education. Alternatively, the employment and social opportunities available to individuals with different levels of education might influence their risk exposure [40,41,42].

Women were more vulnerable to suffering a violent death at home, regardless of the mechanism, as well as on the streets due to physical or chemical mechanisms. In contrast, men died from firearms or injuries caused by sharp objects. This establishes a complex risk profile with sex-specific characteristics, but intersecting with structural elements, such as the region of residence, marginalization, education, and poverty, all of which can be addressed in violence intervention as a public health problem, where women’s vulnerability in family environments makes them more prone to experiencing all types of violence [43,44].

In this way, we found that for some sociodemographic variables, such as age, education, and marital status, along with structural variables like the percentage of the population in poverty and the degree of marginalization in the municipality of residence, there is a common profile of vulnerability between men and women. In contrast, occupation, place of death, and regional risk profiles linked to structural characteristics exhibit unique traits between the sexes. Characterizing these profiles between men and women can aid in the design of preventive strategies and the implementation of local policies to reduce the impact of homicides.

The magnitude of homicides in Mexico, as well as in other parts of the world, has implications at different levels. The traumatic event of losing a loved one or someone close to a social group impacts the incidence of cardiovascular diseases, metabolic disorders, substance abuse, depression, and anxiety, all of which leave a transgenerational footprint and constitute the main causes of morbidity in the country [45,46,47,48].

According to INEGI, most men are “heads of household” (in Mexico, 8 out of 10 families rely on a male provider) and have a higher probability of being married. These men are usually in economically productive ages, which aligns with what the United Nations has reported [10]. These findings indicate that these deaths have a significant impact on economic and social dynamics, limiting social mobility and opportunities to break cycles of violence.

By understanding violence as a phenomenon similar to infectious diseases, which does not occur in isolation in time, space, or people, the possibility of intervening from different health settings in the prevention of deaths is created. This implies acting on affected individuals and the environments where non-fatal events occur, whether in outpatient clinics or emergency areas [49].

There are reports of interventions that have successfully impacted victims and perpetrators of violence to reduce instances of aggression in the community, such as preventive interviews with potential perpetrators, cognitive therapy, and raising awareness of domestic violence issues and gang violence by emergency services [49,50,51,52]. However, these individually implemented strategies have their limitations, so they must be paired with others which seek to impact at the community level, such as improving urban infrastructure, reclaiming public spaces, and engaging the population in local community activities that rebuild the social fabric in regions where violence is most observed [53,54]. Primary care physicians can play a crucial role in creating and implementing interventions focused on reducing violence and its effects since they understand that both victims and perpetrators are members of the community to which they provide care. Collaborative work with family members and leaders of organized civil society can allow for the identification of individuals at risk of participating in the cycle of violence and taking measures to mitigate it [51].

On the other hand, government participation plays a crucial role in curbing homicides. In Mexico, strategies aimed at weakening the recruitment capacity of organized crime would potentially impact the level of homicides but also the over one hundred thousand people who have disappeared in recent years [55,56,57]. According to the Mexico Peace Index 2023, the national rate of organized crime has increased by 64.2% in the last eight years, along with rising levels of corruption and impunity [58], as evidenced by the fact that 93 out of every 100 homicides go unpunished in the country [59]. Therefore, the State needs to assume a greater degree of responsibility to fulfill its role as a guarantor of peace. Furthermore, it is essential that at different levels of government and in the states of the Mexican Republic, the magnitude of the problem is recognized, independent of political and ideological positions, and that strategies based on evidence are proposed for its eradication.

### Limitations

The lack of information related to different study variables represents a limitation in the analysis and the nature of records makes understanding the dynamics of violence challenging, such as the low percentage of records with information on the relationship with the perpetrator of violence or the site of occurrence. Enriching this information could help in better diagnosing such dynamics, particularly domestic violence, which has a higher prevalence in women. Also, the data collected might not fully capture all instances of violent deaths due to the limitations in the reporting systems. Furthermore, the study did not include a spatial correspondence analysis of multiple homicides, which limits the ability to understand patterns related to the geographical proximity of adjacent homicides. The findings might not be generalizable to all regions or demographic groups within Mexico; differences in local contexts, such as the presence of organized crime and law enforcement practices, could impact the patterns and dynamics of homicides.

## 5. Conclusions

Research on homicides in Mexico between 2015 and 2022 reveals a problem of great magnitude and complexity. The results show that homicides affect men considerably; however, women suffer from more adverse conditions and greater vulnerabilities. Additionally, there was an upward trend from 2015 to 2019 in men, while in women, deaths increased from 2015 to 2022 for specific age groups. These disparities underscore the importance of considering gender dimensions in violence prevention and response. Homicides were concentrated in various states of the Mexican Republic, particularly in the North of the country, while occupation, education, marital status, and place of occurrence had significant associations in logistic regression models.

It is fundamental and urgent to implement comprehensive prevention strategies that address the underlying causes of violence, as well as early intervention programs aimed at the most vulnerable groups.

## Figures and Tables

**Table 1:** Sociodemographic characteristics of homicides in Mexico from 2015–2022 (*n* = 229,182).

Characteristics	Men n (%)	Women n (%)	* p
**Place of death**			
Ministry of Health hospitals	21,694 (10.63%)	2231 (8.82%)	<0.001
Social Security hospitals	7883 (3.86%)	961 (3.80%)	0.522
National Defense hospitals	135 (0.06%)	18 (0.07%)	0.872
Private hospitals	2843 (1.39%)	354 (1.40%)	0.964
Home	17,220 (8.44%)	5621 (22.22%)	<0.001
Streets and highways	101,246 (49.65%)	9475 (37.47%)	<0.001
Not specified	52,877 (25.93%)	6624 (26.18%)	0.370
**Marital status**			
Single	85,731 (42.04%)	11,151 (44.10%)	0.045
Married or cohabitation	92,772 (45.49%)	9356 (37.00%)	<0.001
Separated or divorced	8351 (4.09%)	2330 (9.21%)	<0.001
Not specified	17,044 (8.35%)	2447 (9.67%)	0.014
**Education**			
None or elementary	69,142 (33.91%)	8207 (32.45%)	<0.001
Secondary or high school	103,587 (50.80%)	12,464 (49.29%)	0.045
Bachelor’s degree or postgraduate	16,891 (8.28%)	2629 (10.39%)	<0.001
Not specified	14,278 (7.00%)	1984 (7.84%)	0.045
**Occupation**			
Administrative	15,233 (7.47%)	1300 (5.15%)	<0.001
Agriculture	21,229 (10.41%)	315 (1.13%)	<0.001
Commerce	24,625 (12.07%)	3040 (12.02%)	0.811
Security	7650 (3.75%)	742 (2.93%)	<0.001
Technician	63,177 (30.90%)	1346 (5.32%)	<0.001
Unemployed	24,679 (12.10%)	12,574 (49.72%)	<0.001
Not specified	47,305 (23.20%)	5967 (23.50%)	0.159

* χ^2^ analysis was performed to compare frequencies between the variable strata.

**Table 2:** Regional incidence according to sex and age groups.

Age Group (Men)	North Central	Center	North	Northwest	South	National
0–4	0.82	1.10	1.04	0.86	0.53	0.89
5–9	0.86	0.93	0.69	0.66	0.56	0.76
10–14	2.24	2.52	2.58	2.50	1.85	2.32
15–19	36.09	29.21	41.15	44.02	25.27	32.45
20–24	80.50	59.33	88.94	87.56	56.77	69.14
25–29	97.26	68.40	104.46	99.36	64.98	80.50
30–34	97.13	70	107.81	96.04	64.86	81.43
35–39	96.45	64.93	106.77	93.51	67.19	79.64
40–44	84.04	57.26	95.14	81.13	63.31	71.20
45–49	66.32	43.90	73.70	63.10	51.33	55.66
50–54	45.51	34.27	53.57	48.38	43.78	42.56
55–59	34.97	26.54	40.17	34.92	34.29	32.56
60–64	26.99	22.75	33.55	25.48	29.38	27.02
65 or above	18.78	17.34	18.41	20.90	20.22	18.67
Age group (Women)	North Central	Center	North	Northwest	South	National
0–4	0.61	0.78	1.04	0.83	0.46	0.72
5–9	0.67	0.63	0.61	0.78	0.58	0.63
10–14	1.18	1.14	1.25	1.30	0.99	1.14
15–19	7.00	5.28	6.66	6.44	4.42	5.65
20–24	9.26	8.42	12.41	9.08	6.22	8.78
25–29	10.32	8.73	12.11	8.55	6.64	9.05
30–34	8.20	7.98	11.89	7.80	6.22	8.27
35–39	8.34	7.24	10.35	7.13	6.42	7.74
40–44	6.66	6.07	8.26	6.03	5.97	6.51
45–49	5.28	4.72	6.96	4.93	4.66	5.20
50–54	4.30	3.80	5.10	4.15	4.56	4.29
55–59	3.42	3.69	3.72	3.34	4.08	3.71
60–64	3.16	2.86	3.34	2.45	3.09	3.01
65 or above	3.52	3.61	3.33	2.29	3.64	3.47

**Table 3:** OR Individual level sociodemographic characteristics associated with the likelihood of dying from homicide.

Sociodemographic Characteristics	Men OR CI (95%)	*p*	Women OR (CI 95%)	*p*
**Sex**	6.48 (6.39–6.56)	<0.001	Reference	
**Age group in years**				
0–4	Reference		Reference	
5–9	1.66 (1.43–1.92)	<0.001	1.75 (1.48–2.05)	<0.001
10–14	3.72 (3.31–4.18)	<0.001	2.51 (2.18–2.90)	<0.001
15–19	19.03 (17.23–21.08)	<0.001	7.35 (6.54–8.29)	<0.001
20–24	25.38 (23–28.09)	<0.001	8.78 (7.83–9.88)	<0.001
25–29	23.05 (20.89–25.51)	<0.001	7.23 (6.45–8.13)	<0.001
30–34	18.57 (16.84–20.56)	<0.001	5.1 (4.54–5.74)	<0.001
35–39	13 (11.78–14.38)	<0.001	3.26 (2.90–3.67)	<0.001
40–44	8.29 (7.51–9.18)	<0.001	1.763(1.56–1.98)	<0.001
45–49	4.53 (4.10–5.02)	<0.001	0.88 (0.78–0.99)	0.038
50–54	2.45 (2.22–2.71)	<0.001	0.461 (0.40–0.52)	<0.001
55–59	1.27 (1.14–1.40)	<0.001	0.249 (0.21–0.28)	<0.001
60–64	0.71 (0.64–0.79)	<0.001	0.129 (0.11–0.14)	<0.001
65 or above	0.16 (0.14–0.18)	<0.001	0.041 (0.036–0.04)	<0.001
**Occupation**				
Agriculture	Reference		Reference	
Technician	2.89 (2.85–2.94)	<0.001	0.89 (0.794–1.017)	0.085
Security	3.13 (3.05–3.22)	<0.001	1.86 (1.632–2.13)	<0.001
Commerce	2.66 (2.614–2.71)	<0.001	1.09 (0.979–1.23)	0.114
Administrative	1.68 (1.64–1.71)	<0.001	0.37 (0.334–0.42)	<0.001
Unemployed	0.70 (0.69–0.71)	<0.001	0.13 (0.122–0.15)	<0.001
Not specified	3.48 (3.43–3.54)	<0.001	0.93 (0.83–1.04)	0.222
**Scholarity**				
Bachelor’s degree or postgraduate	Reference		Reference	
None or elementary	0.77 (0.76–0.78)	<0.001	0.29 (0.28–0.31)	<0.001
Secondary or high school	2.70 (2.65–2.74)	<0.001	1.74 (1.67–1.81)	<0.001
Not specified	2.09 (2.05–2.14)	<0.001	1.29 (1.22–1.37)	<0.001
**Marital status**				
Married or cohabitation	Reference		Reference	
Single	2.7 (2.67–2.72)	<0.001	2.27 (2.21–2.34)	<0.001
Separated or divorced	0.282 (0.27–0.28)	<0.001	0.22 (0.21–0.24)	<0.001
Not specified	1.69 (1.67–1.72)	<0.001	1.65 (1.58–1.72)	<0.001
**Death location**				
Private hospital	Reference		Reference	
Home	0.539 (0.51–0.56)	<0.001	1.508 (1.35–1.68)	<0.001
Streets and highways	41.77 (40.24–43.38)	<0.001	135.29 (121.89–150.67)	<0.001
Ministry of health hospitals	2.10 (2.02–2.18)	<0.001	2.276 (2.03–2.54)	<0.001
Social security hospital	0.50 (0.48–0.52)	<0.001	0.544 (0.48–0.61)	<0.001
Military hospital	0.35 (0.29–0.41)	<0.001	0.39 (0.23–0.60)	<0.001
Not specified	12.75 (12.28–13.24)	<0.001	20.12 (18.13–22.41)	<0.001
**Structural**				
**Percentage of population with education lagging**	1.018 (1.01–1.01)	<0.001	1.013 (1.011–1.014)	<0.001
**Marginalization degree**				
Very Low	Reference		Reference	
Low	1.37 (1.35–1.38)	<0.001	1.29 (1.25–1.34)	<0.001
Medium	0.88 (0.86–0.89)	<0.001	0.78 (0.73–0.82)	<0.001
High	0.85 (0.84–0.87)	<0.001	0.772 (0.72–0.82)	<0.001
Very high	1.49 (1.45–1.53)	<0.001	1.374 (1.26–1.48)	<0.001
**Percentage of population living in poverty**	1.02 (1.001–1.003)	0.028	1.002 (1.001–1.002)	<0.001

Odds ratios for dying by homicide were estimated by bivariate logistic regression. OR = odds ratio, CI = Confidence interval.

## Data Availability

This study relies on publicly accessible data concerning mortality records, which aligns with our commitment to transparency and open scientific inquiry. The datasets analyzed for the research presented in this article are publicly available and can be accessed through the National Institute of Statistics and Geography (INEGI) at the following link: https://www.inegi.org.mx/programas/mortalidad/#datos_abiertos (accessed on 22 December 2023). These datasets are a comprehensive resource that supports the findings reported in this study. No new data were generated in the course of this research. We consider that the availability of these data enhances the reproducibility and potential for validation of our results and supports future research in the field. The use of publicly available datasets ensures compliance with privacy and ethical standards, facilitating a broader understanding of mortality trends and their implications.

## Supplementary Materials

The following supporting information can be downloaded at: https://www.mdpi.com/article/10.3390/ijerph21050617/s1, Table S1: Number of homicides and incidence rate by year and state of occurrence in men; Table S2: Number of homicides and incidence rate by year and state of occurrence in women; Table S3: Frequencies and percentages of homicides at national and regional level by sex and age group; Table S4: Structural and sociodemographic characteristics associated with homicides by region.
