# Supplementary material for: Characterization of Homicides in Mexico: Analysis of 2015–2022
Source: Int J Environ Res Public Health. 2024 May 13;21(5):617. doi: 10.3390/ijerph21050617 (PMC11121517; doi:10.3390/ijerph21050617)
Supplement: Supplementary file 1 [file ijerph-21-00617-s001.zip › ijerph-2965315-supplementary.pdf]

Table S1: Number of violent deaths and incidence rate by year and state of occurrence in men.

State	2015 n(IR)	2016 n(IR)	2017 n(IR)	2018 n(IR)	2019 n(IR)	2020 n(IR)	2021 n(IR)	2022 n(IR)	Mean n(IR)
Aguascalientes	40 (6.11)	44 (6.59)	71 (10.48)	65 (9.46)	87 (12.48)	76 (10.76)	85 (11.87)	71 (9.79)	539 (9.69)
Baja California Norte	699 (41.57)	954 (56.05)	1816 (104.88)	2361 (134.14)	2059 (115.15)	1923 (105.91)	1962 (106.48)	1767 (94.55)	13541 (94.84)
Baja California Sur	150 (40.91)	217 (57.85)	619 (161.28)	137 (34.92)	62 (15.47)	49 (11.98)	48 (11.51)	35 (8.23)	1317 (42.76)
Campeche	56 (12.36)	78 (16.92)	68 (14.48)	60 (12.55)	67 (13.78)	57 (11.52)	71 (14.12)	79 (15.46)	536 (13.89)
Coahuila	229 (15.29)	206 (13.56)	215 (13.95)	192 (12.29)	218 (13.76)	193 (12.02)	124 (7.62)	126 (7.65)	1503 (12.01)
Colima	190 (53.03)	496 (135.96)	669 (180.08)	550 (145.47)	535 (139.13)	462 (118.2)	434 (109.3)	697 (172.89)	4033 (131.75)
Chiapas	462 (17.76)	461 (17.44)	521 (19.41)	460 (16.89)	535 (19.36)	427 (15.23)	466 (16.39)	410 (14.23)	3742 (17.08)
Chihuahua	1098 (61.14)	1378 (76.07)	1833 (100.13)	2034 (109.99)	2294 (122.87)	2532 (134.38)	2192 (115.31)	1779 (92.81)	15140 (101.58)
Ciudad de México	919 (21.22)	1113 (25.72)	1167 (26.98)	1305 (30.21)	1133 (26.26)	1189 (27.6)	941 (21.89)	630 (14.69)	8397 (24.32)
Durango	193 (21.94)	200 (22.48)	172 (19.15)	125 (13.79)	139 (15.2)	119 (12.91)	127 (13.67)	89 (9.5)	1164 (16.08)
Guanajuato	829 (28.7)	1048 (35.84)	1984 (67.15)	2937 (98.45)	3295 (109.44)	4077 (134.23)	3304 (107.88)	3068 (99.38)	20542 (85.13)
Guerrero	1988 (114.75)	2100 (120.56)	2134 (121.94)	1786 (101.62)	1559 (88.36)	1289 (72.81)	1181 (66.5)	1119 (62.83)	13156 (93.6)
Hidalgo	166 (11.85)	160 (11.27)	236 (16.4)	247 (16.96)	389 (26.39)	318 (21.32)	239 (15.85)	298 (19.55)	2053 (17.44)
Jalisco	934 (23.8)	1030 (25.93)	1239 (30.83)	1988 (48.93)	2042 (49.74)	1770 (42.69)	1767 (42.22)	1425 (33.74)	12195 (37.23)
México	2183 (27.17)	2263 (27.82)	2512 (30.5)	2592 (31.11)	2648 (31.43)	2491 (29.26)	2523 (29.34)	2609 (30.05)	19821 (29.58)
Michoacán	734 (32.55)	1241 (54.53)	1488 (64.85)	1662 (71.87)	2068 (88.77)	1973 (84.09)	2231 (94.44)	1839 (77.34)	13236 (71.05)
Morelos	403 (43.09)	559 (59.01)	558 (58.19)	718 (74.01)	929 (94.69)	892 (89.94)	1035 (103.27)	1046 (103.33)	6140 (78.19)
Nayarit	115 (19.26)	119 (19.61)	376 (60.98)	298 (47.61)	157 (24.72)	140 (21.73)	162 (24.81)	153 (23.12)	1520 (30.23)
Nuevo León	388 (14.91)	528 (19.95)	566 (21.06)	737 (27.03)	855 (30.91)	809 (28.85)	945 (33.26)	1234 (42.88)	6062 (27.35)
Oaxaca	679 (35.19)	653 (33.61)	830 (42.45)	900 (45.76)	977 (49.39)	733 (36.86)	655 (32.77)	636 (31.66)	6063 (38.46)
Puebla	515 (17.05)	608 (19.89)	892 (28.87)	1005 (32.2)	1075 (34.1)	740 (23.25)	651 (20.27)	755 (23.3)	6241 (24.86)
Querétaro	118 (11.59)	114 (10.94)	177 (16.65)	197 (18.18)	180 (16.3)	198 (17.61)	194 (16.96)	158 (13.58)	1336 (15.22)
Quintana Roo	118 (15.34)	159 (20.16)	389 (48.06)	728 (87.74)	632 (74.37)	545 (62.67)	585 (65.81)	572 (62.99)	3728 (54.64)
San Luis Potosí	213 (15.81)	291 (21.42)	433 (31.63)	460 (33.35)	439 (31.6)	705 (50.39)	669 (47.5)	661 (46.64)	3871 (34.79)
Sinaloa	929 (61.94)	1026 (67.67)	1356 (88.63)	978 (63.38)	876 (56.3)	648 (41.32)	449 (28.41)	456 (28.64)	6718 (54.53)
Sonora	471 (32.79)	503 (34.6)	645 (43.79)	796 (53.35)	1127 (74.61)	1342 (87.79)	1603 (103.65)	1386 (88.62)	7873 (64.90)
Tabasco	323 (27)	374 (30.87)	390 (31.82)	463 (37.35)	587 (46.84)	520 (41.06)	383 (29.93)	285 (22.05)	3325 (33.36)

Tamaulipas	493 (28.47)	616 (35.25)	950 (53.9)	865 (48.67)	641 (35.78)	657 (36.39)	513 (28.2)	311 (16.97)	5046 (35.45)
Tlaxcala	63 (9.99)	74 (11.55)	99 (15.26)	118 (17.96)	158 (23.76)	110 (16.35)	128 (18.82)	134 (19.49)	884 (16.64)
Veracruz	819 (20.45)	1085 (26.84)	1564 (38.43)	1281 (31.27)	1252 (30.37)	995 (24)	800 (19.2)	639 (15.26)	8435 (25.72)
Yucatán	47 (4.47)	54 (5.08)	38 (3.53)	48 (4.41)	38 (3.45)	58 (5.21)	43 (3.83)	37 (3.26)	363 (4.15)
Zacatecas	275 (34.92)	455 (57.21)	561 (69.99)	570 (70.59)	559 (68.74)	944 (115.29)	1442 (174.97)	1172 (141.32)	5978 (91.62)

IR= Incidence rate for 100,000 men

Table S2: Number of violent deaths and incidence rate by year and state of occurrence in women.

State	2015 n (IR)	2016 n (IR)	2017 n (IR)	2018 n (IR)	2019 n (IR)	2020 n (IR)	2021 n (IR)	2022 n (IR)	Mean n (IR)
Aguascalientes	7 (1.03)	4 (0.58)	7 (1)	8 (1.13)	7 (0.97)	7 (0.96)	11 (1.49)	11 (1.47)	62 (1.07)
Baja California Norte	102 (6.08)	121 (7.11)	193 (11.15)	252 (14.31)	209 (11.67)	222 (12.2)	240 (12.99)	219 (11.68)	1558 (10.89)
Baja California Sur	21 (5.95)	12 (3.32)	67 (18.09)	20 (5.28)	12 (3.1)	5 (1.26)	7 (1.73)	7 (1.7)	151 (5.05)
Campeche	8 (1.72)	10 (2.12)	6 (1.25)	12 (2.45)	10 (2.01)	6 (1.19)	13 (2.53)	12 (2.3)	77 (1.94)
Coahuila	58 (3.86)	36 (2.36)	34 (2.2)	22 (1.4)	44 (2.76)	32 (1.98)	19 (1.16)	20 (1.21)	265 (2.11)
Colima	24 (6.6)	55 (14.87)	69 (18.35)	72 (18.84)	76 (19.57)	83 (21.05)	48 (11.99)	96 (23.65)	523 (16.86)
Chiapas	65 (2.4)	76 (2.76)	63 (2.25)	45 (1.58)	66 (2.29)	66 (2.26)	58 (1.95)	60 (1.99)	499 (2.18)
Chihuahua	118 (6.48)	150 (8.16)	234 (12.59)	224 (11.92)	267 (14.07)	295 (15.39)	303 (15.65)	231 (11.83)	1822 (12.01)
Ciudad de México	133 (2.81)	144 (3.05)	139 (2.94)	136 (2.88)	141 (2.99)	137 (2.91)	122 (2.59)	103 (2.19)	1055 (2.79)
Durango	8 (0.89)	12 (1.32)	26 (2.82)	19 (2.04)	21 (2.24)	15 (1.58)	10 (1.05)	24 (2.49)	135 (1.80)
Guanajuato	97 (3.19)	121 (3.94)	210 (6.77)	333 (10.63)	372 (11.76)	564 (17.68)	487 (15.13)	499 (15.38)	2683 (10.56)
Guerrero	204 (11.06)	230 (12.4)	192 (10.3)	209 (11.16)	169 (8.99)	144 (7.63)	141 (7.45)	125 (6.58)	1414 (9.44)
Hidalgo	45 (3)	40 (2.63)	47 (3.06)	44 (2.82)	62 (3.93)	41 (2.57)	42 (2.6)	49 (3)	370 (2.95)
Jalisco	132 (3.27)	114 (2.79)	124 (3)	218 (5.22)	233 (5.52)	218 (5.11)	222 (5.16)	174 (4)	1435 (4.25)
México	386 (4.58)	402 (4.72)	449 (5.21)	423 (4.85)	432 (4.9)	395 (4.43)	409 (4.54)	458 (5.04)	3354 (4.78)
Michoacán	79 (3.31)	127 (5.27)	138 (5.69)	163 (6.67)	178 (7.23)	235 (9.48)	256 (10.26)	236 (9.4)	1412 (7.16)
Morelos	44 (4.42)	79 (7.84)	72 (7.07)	80 (7.76)	97 (9.31)	80 (7.6)	96 (9.03)	112 (10.44)	660 (7.93)
Nayarit	10 (1.67)	21 (3.46)	52 (8.43)	35 (5.59)	19 (2.99)	20 (3.1)	27 (4.13)	13 (1.96)	197 (3.91)
Nuevo León	50 (1.92)	79 (2.98)	60 (2.23)	85 (3.12)	95 (3.43)	88 (3.14)	97 (3.41)	150 (5.2)	704 (3.17)
Oaxaca	86 (4.12)	103 (4.9)	116 (5.48)	116 (5.45)	132 (6.16)	106 (4.92)	79 (3.65)	121 (5.56)	859 (5.03)
Puebla	83 (2.55)	93 (2.83)	132 (3.97)	120 (3.57)	117 (3.45)	106 (3.1)	99 (2.87)	94 (2.7)	844 (3.13)
Querétaro	19 (1.81)	15 (1.4)	34 (3.11)	28 (2.51)	25 (2.2)	25 (2.16)	26 (2.21)	25 (2.09)	197 (2.18)
Quintana Roo	20 (2.65)	28 (3.61)	45 (5.65)	87 (10.67)	69 (8.27)	66 (7.73)	74 (8.48)	59 (6.62)	448 (6.71)
San Luis Potosí	36 (2.55)	31 (2.18)	47 (3.28)	53 (3.67)	47 (3.23)	57 (3.89)	76 (5.14)	58 (3.9)	405 (3.48)
Sinaloa	50 (3.3)	79 (5.15)	81 (5.23)	41 (2.63)	50 (3.17)	34 (2.14)	29 (1.81)	34 (2.11)	398 (3.19)

Sonora	62 (4.28)	52 (3.54)	61 (4.1)	60 (3.98)	106 (6.94)	87 (5.63)	139 (8.88)	139 (8.78)	706 (5.76)
Tabasco	32 (2.6)	46 (3.69)	43 (3.41)	46 (3.6)	76 (5.89)	55 (4.21)	45 (3.41)	41 (3.07)	384 (3.73)
Tamaulipas	65 (3.69)	122 (6.85)	132 (7.35)	111 (6.12)	104 (5.69)	71 (3.85)	59 (3.17)	48 (2.56)	712 (4.91)
Tlaxcala	11 (1.66)	10 (1.48)	17 (2.49)	18 (2.61)	25 (3.58)	27 (3.82)	28 (3.91)	16 (2.21)	152 (2.72)
Veracruz	103 (2.43)	131 (3.06)	196 (4.55)	134 (3.09)	159 (3.64)	132 (3)	104 (2.35)	104 (2.34)	1063 (3.05)
Yucatán	9 (0.83)	9 (0.82)	13 (1.18)	9 (0.8)	4 (0.35)	12 (1.05)	7 (0.6)	6 (0.51)	69 (0.76)
Zacatecas	21 (2.58)	76 (9.25)	89 (10.75)	87 (10.42)	72 (8.56)	100 (11.8)	156 (18.27)	151 (17.56)	752 (11.14)

IR= Incidence rate for 100,000 women

Table S3. Frequencies and percentages of homicides at national and regional level by sex and age group.

Age group Men	North Central n (%)	Center n (%)	North n (%)	Northwest n (%)	South n (%)	National n (%)
0-4	55 (0.16)	173 (0.26)	82 (0.16)	27 (0.16)	59 (0.14)	396 (0.19)
5-9	57 (0.16)	149 (0.22)	55 (0.11)	21 (0.12)	63 (0.16)	345 (0.16)
10-14	150 (0.44)	414 (0.63)	207 (0.42)	80 (0.47)	204 (0.51)	1055 (0.51)
15-19	2371 (6.99)	4815 (7.36)	3308 (6.72)	1386 (8.30)	2682 (6.81)	14562 (7.12)
20-24	5108 (15.0)	9689 (14.8)	7081 (14.4)	2660 (15.9)	5728 (14.5)	30266 (14.8)
25-29	5861 (17.3)	10811 (16.5)	7999 (16.2)	2827 (16.9)	6109 (15.5)	33607 (16.4)
30-34	5227 (15.4)	10059 (15.3)	7515 (15.2)	2430 (14.5)	5416 (13.7)	30647 (14.9)
35-39	4561 (13.4)	8343 (12.7)	6732 (13.6)	2115 (12.6)	4939 (12.5)	26690 (13.0)
40-44	3656 (10.7)	6840 (10.4)	5672 (11.5)	1733 (10.3)	4282 (10.8)	22183 (10.8)
45-49	2660 (7.85)	4903 (7.49)	4151 (8.44)	1266 (7.58)	3213 (8.16)	16193 (7.91)
50-54	1590 (4.69)	3369 (5.15)	2638 (5.36)	846 (5.06)	2403 (6.10)	10846 (5.30)
55-59	1026 (3.02)	2186 (3.34)	1626 (3.30)	507 (3.03)	1590 (4.04)	6935 (3.39)
60-64	629 (1.85)	1475 (2.25)	1046 (2.12)	294 (1.76)	1087 (2.76)	4531 (2.21)
65 or above	923 (2.72)	2188 (3.34)	1053 (2.14)	505 (3.02)	1573 (3.99)	6242 (3.05)
**Age group women**	**North Central n (%)**	**Center N (%)**	**North n (%)**	**Northwest n (%)**	**South n (%)**	**National n (%)**
0-4	39 (1.01)	118 (1.26)	80 (1.38)	25 (1.53)	49 (1.01)	311 (1.22)
5-9	43 (1.12)	98 (1.05)	47 (0.81)	24 (1.46)	63 (1.30)	275 (1.08)
10-14	77 (2.00)	180 (1.93)	97 (1.68)	40 (2.44)	106 (2.20)	500 (1.97)
15-19	449 (11.7)	847 (9.09)	513 (8.89)	194 (11.8)	461 (9.57)	2464 (9.71)
20-24	580 (15.1)	1363 (14.6)	945 (16.3)	266 (16.2)	632 (13.1)	3786 (14.9)
25-29	627 (16.3)	1409 (15.1)	898 (15.5)	240 (14.6)	652 (13.5)	3826 (15.0)
30-34	468 (12.1)	1239 (13.3)	833 (14.4)	205 (12.5)	571 (11.8)	3316 (13.0)
35-39	436 (11.3)	1061 (11.3)	683 (11.8)	174 (10.6)	542 (11.2)	2896 (11.4)
40-44	323 (8.41)	841 (9.02)	519 (8.99)	139 (8.51)	466 (9.68)	2288 (9.02)

45-49	234 (6.09)	601 (6.45)	404 (7.00)	104 (6.36)	328 (6.81)	1671 (6.58)
50-54	164 (4.27)	421 (4.51)	257 (4.45)	76 (4.65)	276 (5.73)	1194 (4.70)
55-59	110 (2.86)	344 (3.69)	155 (2.68)	52 (3.18)	209 (4.34)	870 (3.42)
60-64	82 (2.13)	213 (2.28)	110 (1.90)	30 (1.83)	127 (2.63)	562 (2.21)
65 or above	205 (5.34)	580 (6.22)	226 (3.91)	64 (3.91)	331 (6.87)	1406 (5.54)

Table S4. Structural and sociodemographic characteristics associated with homicides by region.

Structural factor (men)	North Central	Center	North	Northwest	South
Percentage of population living in poverty	1.006 (1.005-1.007)	1.011 (1.01-1.011)	1.023 (1.022-1.024)	1.014 (1.013-1.015)	1.002 (1.002-1.003)
Marginazation index	0.138 (0.107_0.178)	0.012 (0.01-0.014)	0.002 (0.001-0.002)	0.028 (0.022-0.036)	0.171 (0.145_0.201)
Education lag	1.033 (1.032-1.034)	1.036 (1.035-1.037)	1.06 (1.05-1.06)	1.048 (1.045-1.051)	0.998 (0.997-1)
**Structural factor (women)**	**North Central**	**Center**	**North**	**Northwest**	**South**
Percentage of population living in poverty	1.007 (1.005-1.009)	1.012 (1.011-1.014)	1.021 (1.018-1.023)	1.018 (1.015-1.022)	1.003 (1.001-1.004)
Marginalization index	0.083 (0.041-0.17)	0.013 (0.008-0.021)	0.004 (0.003-0.007)	0.041 (0.02-0.088)	0.135 (0.087-0.21)
Education lag	1.031 (1.028-1.035)	1.03 (1.027-1.033)	1.05 (1.04-10.05)	1.045 (1.036-1.053)	1 (0.997-1.003)

Bivariate logistic regression models were run, the dependent variable was a dichotomic variable with homicide and other causes as contrasting categories.

OR=odds ratio, CI= Interval confidence
